# Composite Interval Mapping Based on Lattice Design for Error Control May Increase Power of Quantitative Trait Locus Detection

**DOI:** 10.1371/journal.pone.0130125

**Published:** 2015-06-15

**Authors:** Jianbo He, Jijie Li, Zhongwen Huang, Tuanjie Zhao, Guangnan Xing, Junyi Gai, Rongzhan Guan

**Affiliations:** 1 National Key Laboratory for Crop Genetics and Germplasm Enhancement, Jiangsu Collaborative Innovation Center for Modern Crop Production, Nanjing Agricultural University, Nanjing, Jiangsu, China; 2 National Center for Soybean Improvement, Ministry of Agriculture, Nanjing, Jiangsu, China; 3 Key Laboratory of Biology and Genetic Improvement of Soybean, Ministry of Agriculture, Nanjing, Jiangsu, China; 4 Department of Agronomy, Henan Institute of Science and Technology, Collaborative Innovation Center of Modern Biological Breeding, Xinxiang, Henan, China; Zhejiang University, CHINA

## Abstract

Experimental error control is very important in quantitative trait locus (QTL) mapping. Although numerous statistical methods have been developed for QTL mapping, a QTL detection model based on an appropriate experimental design that emphasizes error control has not been developed. Lattice design is very suitable for experiments with large sample sizes, which is usually required for accurate mapping of quantitative traits. However, the lack of a QTL mapping method based on lattice design dictates that the arithmetic mean or adjusted mean of each line of observations in the lattice design had to be used as a response variable, resulting in low QTL detection power. As an improvement, we developed a QTL mapping method termed composite interval mapping based on lattice design (CIMLD). In the lattice design, experimental errors are decomposed into random errors and block-within-replication errors. Four levels of block-within-replication errors were simulated to show the power of QTL detection under different error controls. The simulation results showed that the arithmetic mean method, which is equivalent to a method under random complete block design (RCBD), was very sensitive to the size of the block variance and with the increase of block variance, the power of QTL detection decreased from 51.3% to 9.4%. In contrast to the RCBD method, the power of CIMLD and the adjusted mean method did not change for different block variances. The CIMLD method showed 1.2- to 7.6-fold higher power of QTL detection than the arithmetic or adjusted mean methods. Our proposed method was applied to real soybean (*Glycine max*) data as an example and 10 QTLs for biomass were identified that explained 65.87% of the phenotypic variation, while only three and two QTLs were identified by arithmetic and adjusted mean methods, respectively.

## Introduction

A quantitative trait is usually regarded as complex because of its inheritance mechanism [1]. In the past two decades, unraveling the genetic basis of quantitative traits has become an attractive and challenging research field. Great efforts have been made in quantitative trait locus (QTL) mapping, based on molecular markers, to identify the genetic architecture underlying quantitative phenotypic variation [2–6]. Generally, to effectively map the QTLs of a trait, a proper statistical method, a genetic population, and an efficient experimental design are required both for powerful and accurate QTL mapping.

Numerous statistical methods have been proposed for QTL mapping [7], among which single marker regression is the simplest method, which identifies QTLs by testing the difference between marker group means on the phenotype, using methods such as analysis of variance (ANOVA). The single marker regression approach can only detect QTLs at marker positions, thus requiring an ultra-high density of markers to obtain accurate estimates of QTL locations [8]. Interval mapping (IM) was proposed to map genome-wide QTLs based on linkage maps [9]. The IM method performs a statistical test for a QTL at each genome position between a pair of markers by conditioning on the genotypes of the two flanking markers. However, two or more linked QTLs may affect the mapping in IM, leading to biased estimates of locations and effects of QTLs [10–12]. Based on IM, composite interval mapping (CIM) was proposed to reduce the impact of linkage on QTL under testing and to improve the precision of QTL mapping [12]. More recently, a further refinement was made to reduce the impact of covariate marker selection on CIM, which was designated as inclusive composite interval mapping (ICIM) [13]. Furthermore, various multi-locus model methods based on Bayesian statistical frameworks have also been developed for simultaneously modeling multiple genome-wide QTLs [14–18]. Although Bayesian methods have a number of advantages for QTL mapping, they are usually computationally intensive and rarely easy to use. With the user-friendly computer program Windows QTL Cartographer [19], CIM is currently the most widely used method for QTL mapping in segregating populations derived from bi-parental crosses.

Various types of genetic segregating populations used for QTL mapping may be classified into tentative mapping populations, such as F_2_ and backcross (BC), and permanent mapping populations, such as recombinant inbred lines (RILs) and doubled haploid lines (DH). Genetic experiments with tentative mapping populations may not repeat between years or locations. On the contrary, genetic experiments with permanent mapping population can be repeated and can evaluate genotypes by environment interaction. Thus, permanent mapping populations are currently preferred for QTL mapping as the phenotypes of mapping population individuals can be evaluated in multiple environments with multiple replications.

Both accurate genotype and phenotype data are required for high-resolution QTL mapping. The current genotyping technologies are much more reliable; therefore, obtaining high-quality phenotypic data becomes more important in QTL studies [20, 21]. Appropriate experimental design is critical to reduce the experimental error and phenotype evaluation [22]. For tentative mapping populations, such as F_2_s and BCs, a completely randomized design (CRD) is usually implemented in a phenotyping experiment. However, non-uniform field conditions and lack of replication can result in large experimental errors in CRD, which further reduce the accuracy of QTL mapping. For permanent mapping populations, such as RILs and DHs, randomized complete block design (RCBD) is the most widely used experimental design in QTL mapping. RCBD may reduce the experimental error by increasing replications. However, RCBD is not efficient for local control. Lattice design [22], which has efficient local control, is an excellent experimental design for phenotype evaluation of a genetic population, especially for large sample sizes [23]. In recent years, lattice design has been applied increasingly in QTL mapping [24–28]. Previous mapping methods or software could not handle the original data from lattice design; therefore, the adjusted mean or arithmetic mean of each line were used typically as response variables in the mapping procedure [27, 29–31]. In the present study, a QTL mapping method termed composite interval mapping based on lattice design (CIMLD), which is based on lattice design and the most popularly used CIM method, is proposed. Simulations demonstrated that our model improved the power of QTL detection. Real soybean (*Glycine max*) data analyses were performed as a practical example of the proposed method.

## Materials and Methods

### CIMLD

Following the CIM method [12], the linear model for testing a QTL on a marker interval (*v*, *v*+1) in an RIL population with an experiment incorporating lattice design can be written as:

$$y_{ijk}=\mu +\alpha_{i}+\gamma_{j(i)}+b^{*}x_{k}^{*}+\sum_{l\neq v,v+1}b_{l}x_{kl}+\varepsilon_{ijk},$$ (1)

where *y*_*ijk*_ is the phenotypic observation of the *k*-th (*k* = 1, 2,…, *t*) line in the *j*-th (*j* = 1, 2, …, *s*) block within the *i*-th (*i* = 1, 2,…, *r*) replication, *μ* is the overall mean, *α*_*i*_ is the effect of *i*-th replication, *γ*_j(i)_ is the effect of *j*-th block within *i*-th replication, b* is the effect of the QTL and *x*_k_* is a dummy variable taking a value of 0 or 1 and denotes the genotype of *k*-th line at the QTL, *b*_*l*_ is the effect of *l*-th (*l* = 1, 2, …, *p*) covariate marker representing the background effect and *x*_*kl*_ denotes the genotype of *k*-th line at *l*-th covariate marker, taking a value of 0 or 1. *ε*_*ijk*_ is the random error effect. All effects were regarded as fixed except *γ*_j(i)_ and *ε*_*ijk*_, $\gamma_{j(i)}\sim N(0,\sigma_{b}^{2})$, *ε*_*ijk*_ ~ *N*(0, *σ* ^2^), where $\sigma_{b}^{2}$ is the variance of the block effect within the replication and *σ* ^2^ is the error variance.

The model (1) can be rewritten in matrix notation as

$$\begin{array}{c} y=1_{n}\mu +X_{R}b_{R}+X_{Q}b_{Q}+X_{M}b_{M}+Zu+e\\ =[1_{n}\text{,}X_{R},X_{Q},X_{M}][\mu ;b_{R};b_{Q};b_{M}]+Zu+e\\ =Xb+Zu+e, \end{array}$$ (2)

where **y** denotes the *n*×1 vector of *y*_*ijk*_, *n* is the total number of observations and equals *t*×*r*, **1**_*n*_ is an *n*×1 vector of 1s, **X**_*R*_ is an *n*×*r* design matrix whose *i*-th column is the dummy variable (taking a value 0 or 1) of *α*_*i*_, **b**_*R*_ is *r*×1 vector of *α*_*i*_, **X**_*Q*_ is an *n*×1 design matrix of QTL, **b**_*R*_ is the same as b*, **X**_*M*_ is an *n*×*p* design matrix of covariate markers, **b**_*M*_ is a *p*×1 vector of *b*_*l*_, **Z** is an *n*×*s* design matrix of block, and **u** is a *s*×1 vector of *γ*_j(i)_.

The variance of **y** is

$$V=\text{Var}(Zu+e)=ZZ^{\prime }\sigma_{b}^{2}+I_{n}\sigma^{2}=\sigma^{2}(ZZ^{\prime }\delta +I_{n})=\sigma^{2}\Sigma ,$$ (3)

where **I**_*n*_ is an *n*×*n* identity matrix, $\delta =\sigma_{b}^{2}/\sigma^{2}$, *δ* may be used to measure the variance of a block within the replication; the covariance structure is **Σ** = **ZZ'** *δ*+ **I**.

Then the phenotype is adjusted as

$$y^{*}=\Sigma^{-1/2}y=\Sigma^{-1/2}Xb+\Sigma^{-1/2}(Zu+e)=X^{*}b+e^{*}.$$ (4)

The variance of y* is **Σ** ^-1/2^Var(**Zu** + **e**)**Σ** ^-1/2^ = **I** *σ* ^2^. Thus, when **Σ** is known, model (4) is just a simple linear model and can be used to test a QTL under the framework of CIM. Here, we make use of the error variance and block-within-replication variance parameter estimated from ANOVA of the lattice design to provide an estimate of *δ*. Then, the estimate of **Σ** can be obtained according to eq (3).

The QTL genotype is generally unknown in QTL mapping. In the standard IM method, conditional probabilities for the QTL genotypes were calculated according the genotype of two flanking markers and incorporated into a mixture of normal distributions [9]. In the present study, multiple versions of the complete QTL genotype were imputed based on the hidden Markov model technology in the R/qtl package [32, 33]. Thus, the model (4) can be solved using standard statistical methods for simple linear regression [34]. Although an *F*-test can be used to test a QTL, we used the traditional logarithm of odds (LOD) score as a convenient statistical test [32]:

$$\text{LOD}=\frac{n}{2}{\text{log}}_{10}(RSS_{0}/RSS_{1}),$$ (5)

where *RSS* is the residual sum of squares; the subscript 1 indicates a linear model including the QTL effects and 0 indicates the null model without the QTL effect. There are multiple versions of the QTL genotype; therefore, the LOD scores at a particular position are averaged to obtain an appropriate combined LOD score for QTL detection [35].

### Simulated data

The genotype data of a RIL population comprising 196 lines were simulated using the R/qtl package [32]. The genome consisted of five chromosomes, each of 150 cM in length and with 16 evenly distributed markers. Ten QTLs (represented by Q1–Q10) were simulated with the same positions and effects as used by Zeng [12]. Three QTLs were located on each of the first three chromosomes, one QTL on the fourth chromosome and no QTLs on chromosome five. The positions and effects of QTL were 16 cM, 48 cM, 108 cM and 0.42, 0.75, 0.58 for the first chromosome;, 3 cM, 43 cM, 77 cM and 1.02, −1.23, −1.26 for the second chromosome; 33 cM, 68 cM, 129 cM and −0.46, 1.61, 0.88 for the third chromosome; and 26 cM and 0.74 for the fourth chromosome, respectively. Furthermore, 100 polygenes with effects drawn from *U* (0, 0.1) were simulated to make the simulated quantitative trait more natural. The genotypic values for each line were obtained as the sum of the QTL and polygene effects.

Simulated phenotype data for a 14×14 simple lattice design experiment with two replications were generated. The replication effect was set to 2. The random error effect was drawn from a normal distribution with a mean of zero and variance of Var(g)×(1/*h* ^2^ − 1), where Var(g) is the total genetic variance and *h* ^2^ is total heritability, and was fixed as 0.7. The block-within-replication effect was drawn from a normal distribution with a mean of zero and variance of Var(e)×*δ*, where Var(e) is the error variance and *δ* is the same as defined in model (3), and was set to 0.5, 1, 5, and 10, respectively. One hundred replications were performed for each scenario.

### Performance analysis

Our proposed CIMLD method was compared with two other mapping strategies: the standard CIM method with the arithmetic mean as the response (referred as RCBD hereinafter) and the standard CIM method with the adjusted mean as the response (referred as AMLD hereinafter). The threshold for the LOD score was determined by a permutation test [36] with 1000 replications or a predefined empirical value of 2.5 [19]. The false discovery rate (FDR) and the power of QTL detection were calculated and used for performance comparisons. A detected QTL was considered a false positive if none of the predefined QTLs were found within a 10 cM window, centered at the detected QTL. A predefined QTL was considered as detected if at least one QTL was found within a 10-cM window, centered at the true QTL. Standard CIM analysis was performed using the *cim* function of R/qtl [32].

### Soybean data set

A RIL population of soybean comprising 184 lines derived from the cross between cultivars Kefeng No.1 and Nannong1138-2 was planted in a 14×14 simple lattice design, with two replications, for 2 years, in 2005 and 2006, in the National Center of Soybean Experiment, Jiangsu, Nanjing Agricultural University, China [37, 38]. The biomass of each RIL was obtained by measuring the dry weight of all plants in each plot. The linkage map of the population was initially constructed from 452 markers and was refined recently [38, 39]. The new map contains 834 molecular makers covering 2308 cM in 24 linkage groups.

### Statistical analysis

A random effects model was used for ANOVA of biomass and the phenotypic value for the *i*-th environment, *j*-th replication, and *l*-th genotype was expressed as *y*_*ijkl*_ = *μ* + *e*_*i*_ + *α*_j(i)_ + *γ*_k(ij)_ + *g*_*l*_ + (*ge*)_*il*_ + *ε*_*ijkl*_, where *y*_*ijkl*_ is the phenotypic observation of the *l*-th line in the *k*-th block within the *i*-th environment and *j*-th replication, *μ* is the overall mean, *e*_*i*_ is the effect of *i*-th environment, *α*_*j*_ is the effect of *j*-th replication, *γ*_k(ij)_ is the effect of *k*-th block within *i*-th environment and *j*-th replication, *g*_*l*_ is the effect of *l*-th line, (*ge*)_*il*_ is genotype by environment interaction effect, and *ε*_*ijkl*_ is random error. All effects were regarded as fixed except *γ*_k(ij)_ and *ε*_*ijkl*_, *γ*_k(ij)_ ~ *N*(0,$\sigma_{b}^{2}$), *ε*_*ijkl*_ ~ *N*(0, *σ* ^2^), where $\sigma_{b}^{2}$ is the variance of the block effect within the replication and *σ* ^2^ is the error variance.

The computation for ANOVA was performed by using the GLM procedure in the SAS/STAT software [40]. Trait heritability of biomass was estimated as $h^{2}=\sigma_{g}^{2}/[\sigma_{g}^{2}+\sigma_{ge}^{2}/s+\sigma^{2}/(s\cdot r)]$, where $\sigma_{g}^{2}$, $\sigma_{ge}^{2}$ and σ^2^ are genotype, genotype by environment interaction and error variance estimated from expected mean squares in ANOVA, respectively, and *s* is the number of environments and *r* is the number of replications [41].

## Results

### Simulation

Researchers have realized that it is necessary to adopt a lattice design to control experimental errors. However, the lack of methods to analyze the full data from a lattice design for QTL mapping caused some previous reports to use the arithmetic means of genotypes as response variables to map QTLs with the CIM method, although the experimental design was lattice. In that case, the experimental design was actually equivalent to RCBD. In other reports, adjusted means of genotypes of the lattice design were used as response variables for QTL mapping with the CIM method. Here, we have described a model (CIMLD) for QTL mapping based on a lattice design. To compare the three strategies, we simulated four error conditions of block-within-replication relative to error variance of the model.

**Fig 1:**
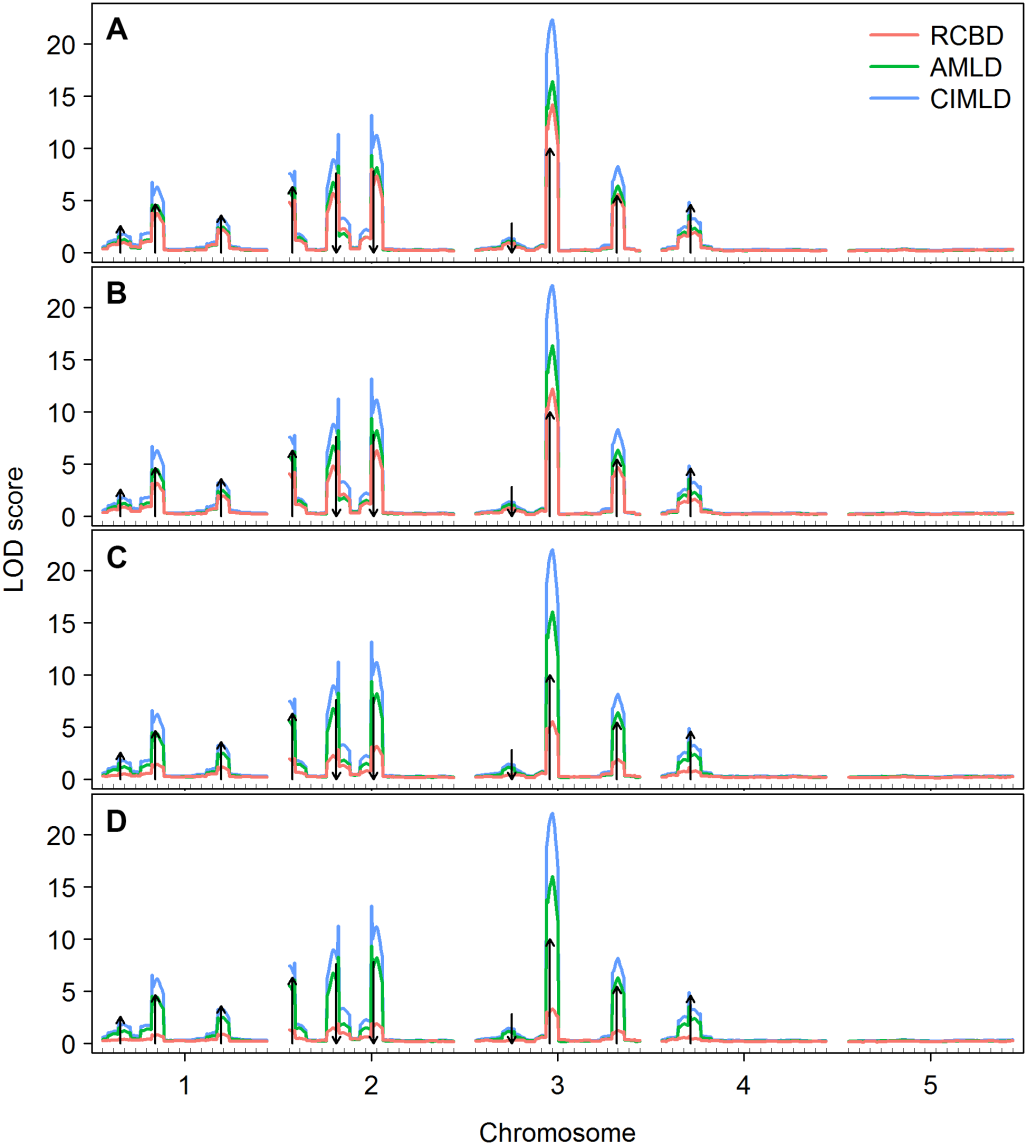
Average LOD scores across 100 replications under different block variances. The arrow size and direction represent the approximate effect size and direction of a QTL, respectively. (A) *δ* = 0.5; (B) *δ* = 1; (C) *δ* = 5; (D) *δ* = 10. *δ* represents the size of the block variance and is defined as $\delta =\sigma_{b}^{2}/\sigma^{2}$, where *σ* ^2^ is the error variance and $\sigma_{b}^{2}$ is the variance of a block within the replication.

**Fig 2:**
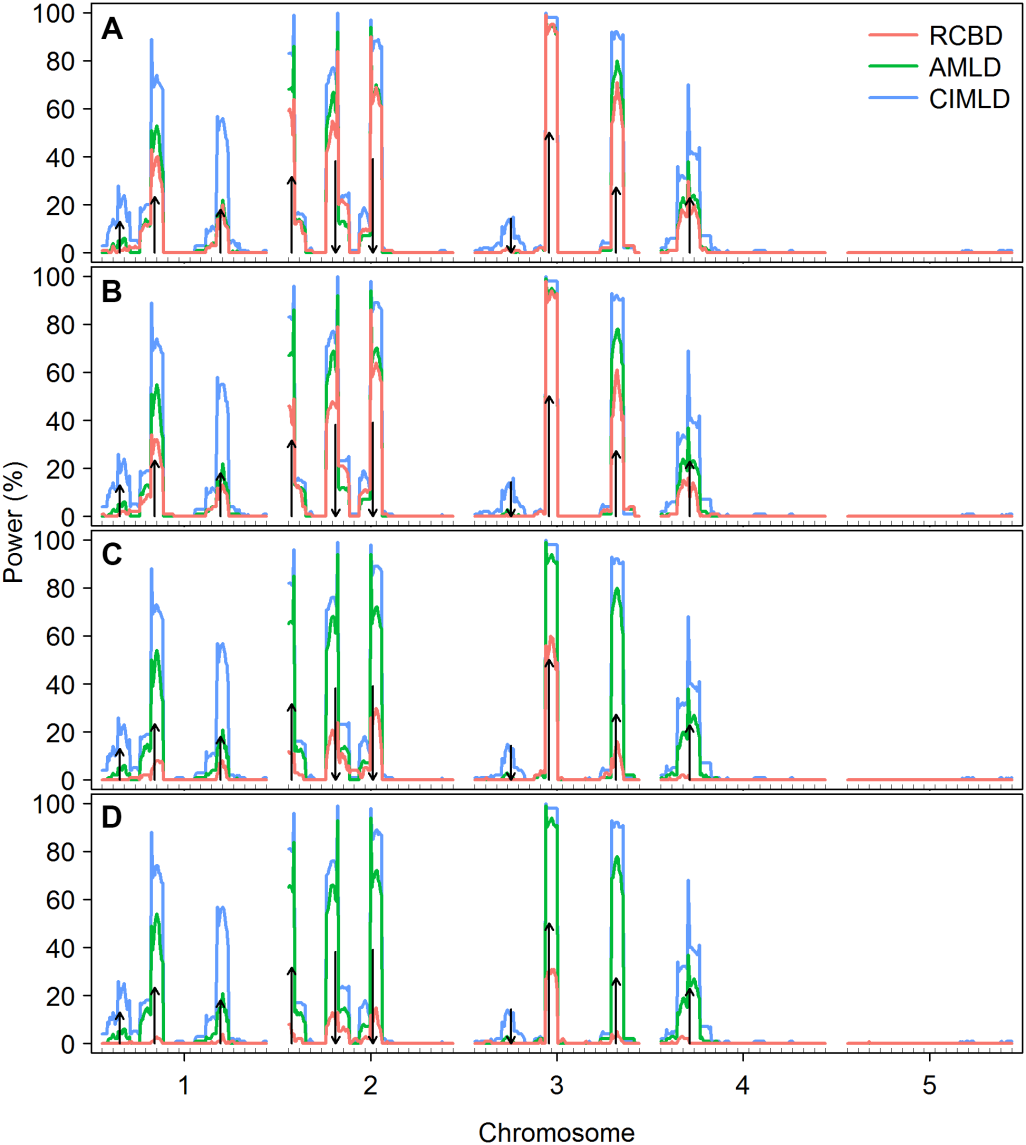
Pointwise power under different block variances. The arrow size and direction represent the approximate effect size and direction of a QTL, respectively. (A) *δ* = 0.5; (B) *δ* = 1; (C) *δ* = 5; (D) *δ* = 10. *δ* represents the size of the block variance and is defined as $\delta =\sigma_{b}^{2}/\sigma^{2}$, where *σ* ^2^ is the error variance and $\sigma_{b}^{2}$ is the variance of a block within the replication. Pointwise power was calculated as the proportion of replications whose LOD score at each testing position was greater than the threshold determined by a permutation test with 1000 replications.

The genome-wide average LOD score profiles under the four block variance conditions (Fig 1) showed that as the block variance increased, the LOD score of the RCBD method decreased rapidly, especially when the block variance was large, e.g. *δ* = 10, where the genome-wide LOD score decreased to almost zero, indicating a rapid loss in power. There is no direct correspondence between the size of the LOD score and power; therefore, we calculated the frequency that a LOD score was greater than the threshold from a permutation test among the 100 replications at each genome position. The pointwise power analysis (Fig 2) confirmed that the RCBD method suffered a high false-negative rate with large block variance. In contrast, the LOD scores of AMLD and CIMLD methods were not sensitive to block variance and stayed stable across the four block conditions, as expected. However, the CIMLD method was likely to be more powerful than AMLD, because the LOD score was generally greater than that of AMLD at the predefined QTL locations in all block conditions. The pointwise power analysis further showed that CIMLD method had a higher power peak at the predefined QTL locations than the AMLD method, especially for small effect QTL locations, such as Q1, Q3, and Q7. Furthermore, clear peaks were observed only around most of the predefined QTL in all the three methods under all block conditions, indicating good accuracy for all three mapping methods for the different block variance conditions.

**Fig 3:**
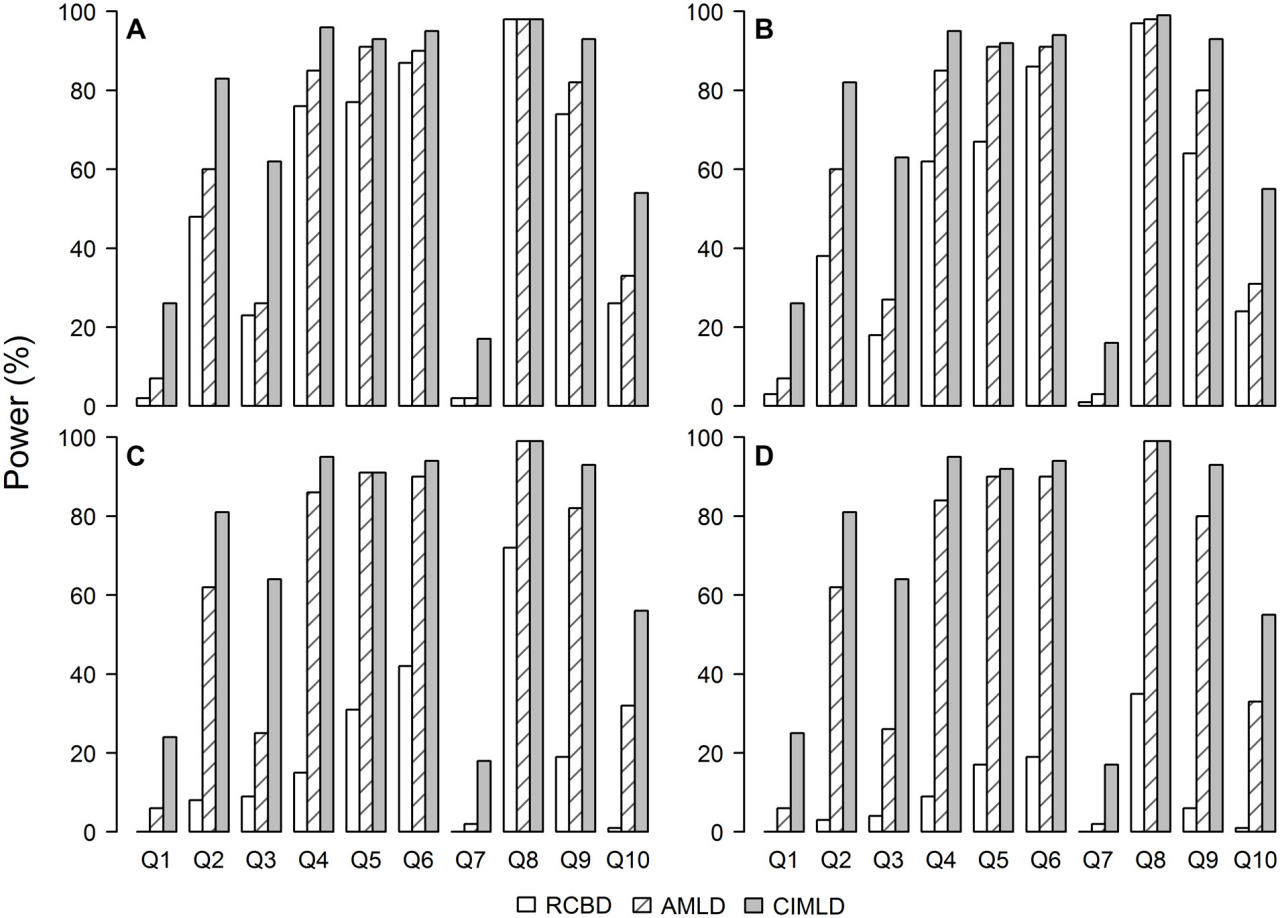
The power of QTL detection for each QTL under different block variances. (A) *δ* = 0.5; (B) *δ* = 1; (C) *δ* = 5; (D) *δ* = 10. *δ* represents the size of the block variance and is defined as $\delta =\sigma_{b}^{2}/\sigma^{2}$, where *σ* ^2^ is the error variance and $\sigma_{b}^{2}$ is the variance of a block within the replication. For the purpose of computing the detection power, a predefined QTL counted as detected if at least one QTL was found within a 10 cM window centered at the true QTL. A permutation test with 1000 replications was used to determine the threshold.

The power of each QTL detection (Fig 3) showed that with increasing block variance, the power of RCBD method decreased rapidly, while the power of AMLD and CIMLD method stayed stable. For all block variances, the power of the RCBD method was generally less than AMLD and CIMLD, especially for large block variances: a large power gap was observed. This indicated that the lack of error control of the RCBD method resulted in a bad performance for QTL mapping. The CIMLD method showed a higher power than the AMLD method for most of the QTL, except for Q5, Q6, and Q8, where the AMLD method had a comparable power to the CIMLD method. For those QTLs with small effects, especially at Q3, the power of the CIMLD method was approximately 40% greater than that for AMLD. The results indicated that the CIMLD method is much more powerful than AMLD, especially for small-effect QTLs.

The FDR and overall power of the three methods were investigated. The FDR for each method (Table 1) was in a reasonable range and was generally less than 1% either for the permutation test or an empirical threshold of 2.5. With increasing = block variance, the FDR of each method remained relatively stable. This indicated that large block variances would not cause an increase in FDR for all three methods.

**Table 1:** False discovery rate (FDR) of QTL detection under different block variances and thresholds.

*δ*	Permutation test			LOD > 2.5		
	RCBD	AMLD	CIMLD	RCBD	AMLD	CIMLD
0.5	0.00508	0.00518	0.00788	0.00698	0.00811	0.00788
1	0.00452	0.00518	0.00667	0.00752	0.00811	0.00667
5	0.00333	0.00500	0.00756	0.01183	0.00911	0.00756
10	0.00500	0.00500	0.00756	0.01917	0.00911	0.00756

*δ* represents the size of the block variance and is defined as $\delta =\sigma_{b}^{2}/\sigma^{2}$, where *σ* ^2^ is the error variance and $\sigma_{b}^{2}$ is the variance of a block within the replication. For the purpose of computing the detection power and FDR, a predefined QTL was counted as detected if at least one QTL was found within a 10 cM window centered at the true QTL, and a detected QTL was considered as a false positive if none of the predefined QTLs were found within a 10-cM window centered at the detected QTL. A permutation test was performed with 1000 replications.

The overall power of QTL detection (Table 2) showed that the RCBD method was very sensitive to the size of block variance. With the increase of block variance, the power of the RCBD method decreased from 51.3% to 9.4%. The power of CIMLD and AMLD methods were both higher than the RCBD method under all four block conditions. The power of the three methods may be ordered as CIMLD > AMLD > RCBD. In contrast to the RCBD method, the power of CIMLD and AMLD did not change for different block variances.

**Table 2:** Overall power of QTL detection under different block variances and thresholds.

*δ*	Permutation test (%)			LOD > 2.5 (%)		
	RCBD	AMLD	CIMLD	RCBD	AMLD	CIMLD
0.5	51.3	57.4	71.7	63.2	67.7	73.2
1	46.0	57.3	71.5	59.0	67.6	73.1
5	19.7	57.5	71.5	35.3	67.8	73.3
10	9.4	57.2	71.5	20.4	67.6	73.3

*δ* represents the size of the block variance and is defined as $\delta =\sigma_{b}^{2}/\sigma^{2}$, where *σ* ^2^ is the error variance and $\sigma_{b}^{2}$ is the variance of a block within the replication. For the purpose of computing the detection power, a predefined QTL was counted as detected if at least one QTL was found within a 10-cM window centered at the true QTL. A permutation test was performed with 1000 replications.

The power of RCBD and AMLD methods under the permutation test was generally less than the empirical threshold of 2.5; however, the CIMLD method was more stable for the two-threshold strategy. The power of the CIMLD method was approximately 14% higher than AMLD in case of the permutation test and approximately 6% higher under an empirical threshold of 2.5. This was because the LOD score of the CIMLD method was much greater than those of the RCBD and AMLD methods (Fig 1), and the threshold from the permutation test was generally greater than 2.5.

Thus, the CIMLD method has a higher power of QTL detection because an appropriate model is implemented and the experimental error is effectively controlled.

### Application to a real soybean data set

Soybean biomass data of a RIL population were used as an example of our proposed CIMLD method (S1 Fig). ANOVA for biomass showed that that the genotype variance was significant and the line-by-environment interaction was also significant (S1 Table); however, the *F* value of the statistical test for the interaction was much less than that of main line effects, thus the phenotypic values of the lines agreed well across environments relative to the variation of the genotypic effect. The estimated heritability of biomass was approximately 82.1%.

To analyze the 2-year soybean data for QTL mapping, a modification of model (1) was needed to include the QTL by environment interaction effect. Although our previous model for QTL mapping did not explicitly take environment factors into account, such environment factors can be treated as fixed effects and modeled as covariates without any further assumptions. Accordingly, the resulting modified version of model (2) can be expressed as **y** = **1**_n_ *μ* + **X**_E_ **b**_E_ + **X**_R_ **b**_R_ + **X**_Q_ **b**_Q_ + **X**_QE_ **b**_QE_ + **X**_M_ **b**_M_ + **Zu** + **e**, where **X**_E_ is the design matrix of the environment effect **b**_E_ and **X**_QE_ is the design matrix of a QTL by the environment interaction effect **b**_QE_. Thus, the CIMLD method with genotype by environment interaction was employed to map QTLs for biomass.

**Fig 4:**
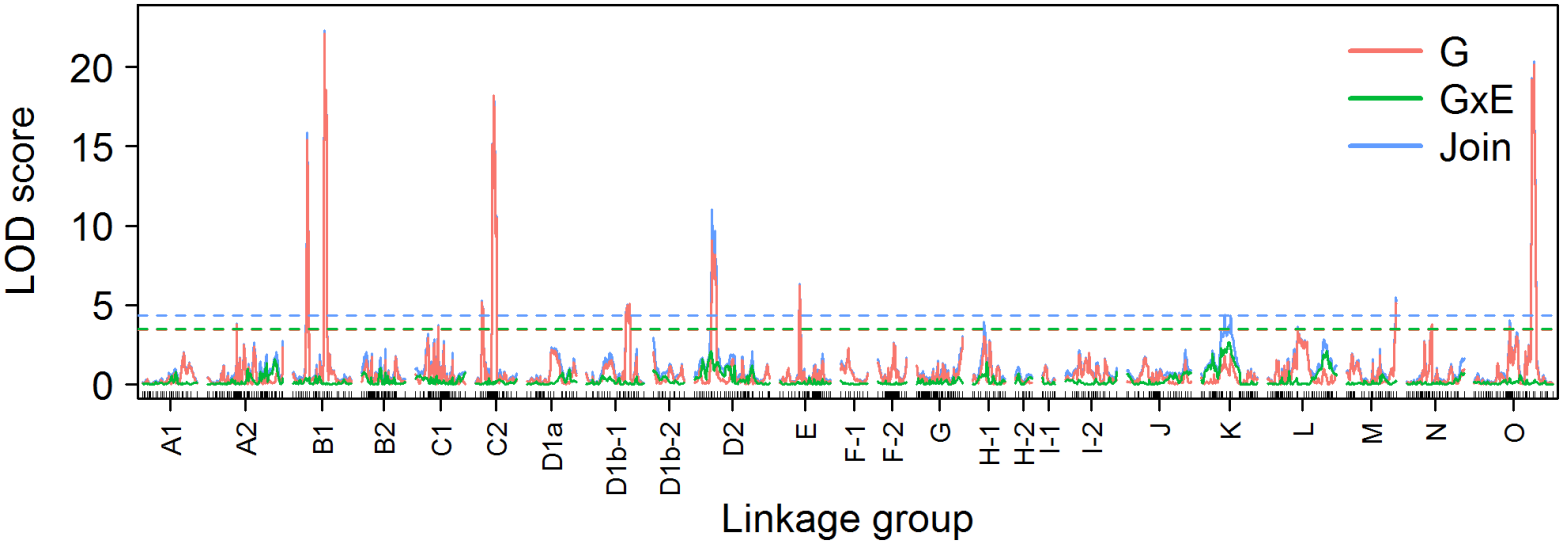
Genome-wide LOD profile for biomass in a population of 184 soybean RILs. G, LOD score of the additive effect; GxE, LOD score of the additive-environment interaction effect; Join, LOD score of the joint additive and additive-environment interaction effects. Dashed lines are thresholds obtained by a permutation test with 1000 replications.

The LOD profile of biomass (Fig 4) showed clear peaks on linkage groups B1, C2, D2, E, and O. The genome-wide thresholds were 4.09, 4.02, and 4.36 for the RCBD, AMLD, and CIMLD methods at a genome-wide significance level of 0.05 by permutation test with 1000 replications, respectively. The RCBD method identified three QTLs located on B1 at 63 cM, C2 at 35.2 cM, and O at 118 cM, respectively. The AMLD method identified two QTLs located on C2 at 35 cM and O at 117 cM.

By contrast, the CIMLD method identified 10 additive QTLs for biomass distributed on eight linkage groups with a total narrow-sense heritability of 65.8%, which are summarized in Table 3. The heritability of the single QTLs ranged from 2.0% to 16.7%, and four large effect QTLs, located on linkage group B1 at 28 cM and 62 cM, C2 at 35.2 cM, and O at 117 cM, were observed with a total heritability of 46.1%. Although no additive-environment QTLs were detected, the additive environment effects of QTLs located on linkage group D2 at 34 cM and K at 46 cM were relatively large, showing a weak environment interaction signal. As predicted by the simulation results, the CIMLD method had a higher power than the RCBD and AMLD method; we identified all the three QTLs detected by RCBD and AMLD (the positions were the same or within 1 cM). The three common QTLs all belonged to the four large-effect QTLs. The effects of newly identified QTLs are relatively small, except for a QTL on B1 at 28 cM, whose heritability was 11.3%.

**Table 3:** QTLs detected for the biomass trait in a population of 184 soybean RILs.

LG	Marker interval, Length (cM)	Support interval (cM)	Position (cM)	LOD	*a*	*ae*	*h* ^2^ (%)
B1	GMpti_D—Sat_247, 4.0	27.7–29.0	28.0	15.87	71.14	-5.35	11.3
B1	A333I—A118T, 2.9	61.0–62.3	62.0	22.28	63.70	6.37	9.2
C2	LI26T—Satt286, 3.4	12.0–15.7	13.0	5.27	-42.54	-3.28	4.1
C2	Satt365, -	34.0–35.7	35.2	18.20	87.84	6.96	16.7
D1b-1	GNT007—Satt698, 4.9	76.0–87.0	84.0	5.11	44.42	0.42	4.5
D2	Satt135—Sat_277, 4.9	33.0–37.0	34.0	11.00	45.54	23.03	4.8
E	GNB080, -	37.0–40.0	37.9	6.33	-29.91	-0.32	2.0
K	Sat_363—GNE097b, 7.3	37.0–61.0	46.0	4.37	29.26	24.69	2.0
M	Satt655—GNE042, 6.1	95.0–97.0	97.0	5.51	32.08	2.44	2.3
O	LE23T—GNE035, 3.1	116.7–118.0	117.0	20.33	-62.55	7.70	8.9

LG, linkage group; *a*, additive effect in kg hm^-2^; *ae*, additive-environment interaction effect in kg hm^-2^; *h* ^2^, narrow-sense heritability. The support interval of a QTL is the interval in which the LOD score is within 1.5 units of its maximum.

## Discussion

Large numbers of permanent population lines are generally required in QTL mapping, and experimental fields or space need to be enlarged accordingly, resulting in larger experimental errors with the increase of lines or genotypes. As such, adopting an experimental design suitable for large numbers of genotypes is critical to control the experimental error. However, most researchers who used the RCBD method for QTL mapping ignored error control. As shown by the simulation results, the RCBD method used in most QTL mappings with permanent populations has a low power of QTL detection because of its lack of local control. Thus, it should be emphasized that error control in QTL mapping is very important for increasing the power of QTL detection and the accuracy of QTL effect estimation. Furthermore, we believe that error control is also important in association mapping, because the error not only affects the estimation of a QTL’s position, but also its effects.

In a lattice design, environmental errors can be decomposed into block-within-replication error and random error, which is the residual of the lattice design model. The CIMLD method is beneficial to QTL studies because of its better error control. The previous QTL mapping based on lattice design mis-specified the model. One mis-specified model, which was actually an RCBD method in this study, has a larger error variance than the CIMLD method, which led to a lower detection power. Another mis-specified model, redefined as the AMLD method, takes block effect into account, and uses adjusted means of genotypes as response variables. AMLD obviously improved the QTL detection power; however, it ignored partial dependence of phenotypic values, caused by random block effect in the lattice design. It is this dependence that violated the hypothesis of independence of observations in the CIM approach, and thus lowered the QTL detection power relatively to the CIMLD method. Our proposed CIMLD method directly modeled the full phenotypic data of the lattice design for QTL mapping, and the simulation showed it to have a good performance in terms of power and FDR.

Our CIMLD implementation is based on CIM and a multiple imputation framework, but could be extended easily to other, more sophisticated statistical methods for QTL mapping, such as ICIM [13], Bayesian LASSO [16] and two-dimensional genome scan to identify epistasis. Furthermore, the concept can be applied to other widely used experimental designs for efficient error control, such as the block-in-replication design, replication-in-block design, and other incomplete block designs [42]. Our method is also applicable to association mapping with pure line populations, because lattice design is very suitable for phenotyping of a large sample.

## Conclusions

Error control is very important for accurate mapping of quantitative traits. Even if the experimental design is implemented for efficient error control, an appropriate linear model is also needed to achieve powerful QTL mapping. Our composite interval mapping method, based on lattice design, was demonstrated to be more powerful for QTL mapping because of its better error control. The concept presented in the current study may be extended to other statistical methods or experimental designs for QTL mapping. An R language implementation of our proposed method has been made publicly available at https://github.com/hjbreg/cimld.

## Supporting Information

S1 Fig The histogram of the biomass trait in a population of 184 soybean RILs. Biomass was measured in kg hm^-2^.
(TIFF)

S1 Table Analysis of variance for the biomass trait in a population of 184 soybean RILs. (DOCX)

## References

[1] Lander ES, Schork NJ. Genetic dissection of complex traits. Science. 1994;265(5181):2037–2048. 8091226 10.1126/science.8091226

[2] Mackay TF. The genetic architecture of quantitative traits. Annu Rev Genet. 2001;35(1):303–339. 11700286 10.1146/annurev.genet.35.102401.090633

[3] Flint J, Valdar W, Shifman S, Mott R. Strategies for mapping and cloning quantitative trait genes in rodents. Nat Rev Genet. 2005;6(4):271–286. 15803197 10.1038/nrg1576

[4] Nordborg M, Weigel D. Next-generation genetics in plants. Nature. 2008;456(7223):720–723. 10.1038/nature07629 19079047

[5] Mackay TF, Stone EA, Ayroles JF. The genetics of quantitative traits: challenges and prospects. Nat Rev Genet 2009;10(8):565–577. 10.1038/nrg2612 19584810

[6] Fu W, O'Connor TD, Akey JM. Genetic architecture of quantitative traits and complex diseases. Curr Opin Genet Dev. 2013;23(6):678–683. 10.1016/j.gde.2013.10.008 24287334 PMC6764439

[7] Xu S. Principles of statistical genomics. New York: Springer; 2013.

[8] Doerge RW, Zeng ZB, Weir BS. Statistical issues in the search for genes affecting quantitative traits in experimental populations. Stat Sci. 1997;12(3):195–219.

[9] Lander ES, Botstein D. Mapping mendelian factors underlying quantitative traits using RFLP linkage maps. Genetics. 1989;121(1):185–199. 2563713 10.1093/genetics/121.1.185 PMC1203601

[10] Jansen RC. Interval mapping of multiple quantitative trait loci. Genetics. 1993;135(1):205–211. 8224820 10.1093/genetics/135.1.205 PMC1205619

[11] Zeng ZB. Theoretical basis for separation of multiple linked gene effects in mapping quantitative trait loci. Proc Natl Acad Sci U S A. 1993;90(23):10972–10976. 8248199 10.1073/pnas.90.23.10972 PMC47903

[12] Zeng ZB. Precision mapping of quantitative trait loci. Genetics. 1994;136(4):1457–1468. 8013918 10.1093/genetics/136.4.1457 PMC1205924

[13] Li H, Ye G, Wang J. A modified algorithm for the improvement of composite interval mapping. Genetics. 2007;175(1):361–374. 17110476 10.1534/genetics.106.066811 PMC1775001

[14] Sillanpaa MJ, Arjas E. Bayesian mapping of multiple quantitative trait loci from incomplete inbred line cross data. Genetics. 1998;148(3):1373–1388. 9539450 10.1093/genetics/148.3.1373 PMC1460044

[15] Yi N, George V, Allison DB. Stochastic search variable selection for identifying multiple quantitative trait loci. Genetics. 2003;164(3):1129–1138. 12871920 10.1093/genetics/164.3.1129 PMC1462611

[16] Yi N, Xu S. Bayesian LASSO for quantitative trait loci mapping. Genetics. 2008;179(2):1045–1055. 10.1534/genetics.107.085589 18505874 PMC2429858

[17] Mutshinda CM, Sillanpaa MJ. Extended Bayesian LASSO for multiple quantitative trait loci mapping and unobserved phenotype prediction. Genetics. 2010;186(3):1067–1075. 10.1534/genetics.110.119586 20805559 PMC2975286

[18] Cai X, Huang A, Xu S. Fast empirical Bayesian LASSO for multiple quantitative trait locus mapping. BMC Bioinformatics. 2011;12:211 10.1186/1471-2105-12-211 21615941 PMC3125263

[19] Wang S, Basten CJ, Zeng ZB. Windows QTL Cartographer 2.5. Department of Statistics, North Carolina State University, Raleigh, NC. Available: http://statgen.ncsu.edu/qtlcart/WQTLCart.htm.

[20] Cobb JN, Declerck G, Greenberg A, Clark R, McCouch S. Next-generation phenotyping: requirements and strategies for enhancing our understanding of genotype-phenotype relationships and its relevance to crop improvement. Theor Appl Genet. 2013;126(4):867–887. 10.1007/s00122-013-2066-0 23471459 PMC3607725

[21] Houle D, Govindaraju DR, Omholt S. Phenomics: the next challenge. Nat Rev Genet. 2010;11(12):855–866. 10.1038/nrg2897 21085204

[22] Hinkelmann K, Kempthorne O. Design and analysis of experiments, introduction to experimental design, vol. 1, 2nd ed New Jersey: John Wiley & Sons; 2007.

[23] Yates F. A new method of arranging variety trials involving a large number of varieties. J Agr Sci. 1936;26(03):424–455.

[24] Dixit S, Huang BE, Sta Cruz MT, Maturan PT, Ontoy JC, Kumar A. QTLs for tolerance of drought and breeding for tolerance of abiotic and biotic stress: an integrated approach. PLoS One. 2014;9(10):e109574 10.1371/journal.pone.0109574 25314587 PMC4196913

[25] Dixit S, Swamy BP, Vikram P, Ahmed HU, Sta Cruz MT, Amante M, et al Fine mapping of QTLs for rice grain yield under drought reveals sub-QTLs conferring a response to variable drought severities. Theor Appl Genet. 2012;125(1):155–169. 10.1007/s00122-012-1823-9 22361948

[26] Hung HY, Browne C, Guill K, Coles N, Eller M, Garcia A, et al The relationship between parental genetic or phenotypic divergence and progeny variation in the maize nested association mapping population. Heredity. 2012;108(5):490–499. 10.1038/hdy.2011.103 22027895 PMC3330692

[27] Strigens A, Schipprack W, Reif JC, Melchinger AE. Unlocking the genetic diversity of maize landraces with doubled haploids opens new avenues for breeding. PLoS One. 2013;8(2):e57234 10.1371/journal.pone.0057234 23451190 PMC3579790

[28] Wen Z, Tan R, Yuan J, Bales C, Du W, Zhang S, et al Cregan P et al: Genome-wide association mapping of quantitative resistance to sudden death syndrome in soybean. BMC Genomics. 2014;15(1):809. 25249039 10.1186/1471-2164-15-809 PMC4189206

[29] Lin Y, Gnanesh BN, Chong J, Chen G, Beattie AD, Mitchell Fetch JW, et al A major quantitative trait locus conferring adult plant partial resistance to crown rust in oat. BMC Plant Biol. 2014;14:250 10.1186/s12870-014-0250-2 25260759 PMC4181729

[30] Bansal U, Bariana H, Wong D, Randhawa M, Wicker T, Hayden M, et al Molecular mapping of an adult plant stem rust resistance gene Sr56 in winter wheat cultivar Arina. Theor Appl Genet. 2014;127(6):1441–1448. 10.1007/s00122-014-2311-1 24794977

[31] Barriere Y, Mechin V, Lefevre B, Maltese S. QTLs for agronomic and cell wall traits in a maize RIL progeny derived from a cross between an old Minnesota13 line and a modern Iodent line. Theor Appl Genet. 2012;125(3):531–549. 10.1007/s00122-012-1851-5 22437492

[32] Broman KW, Wu H, Sen S, Churchill GA. R/qtl: QTL mapping in experimental crosses. Bioinformatics. 2003;19(7):889–890. 12724300 10.1093/bioinformatics/btg112

[33] Broman KW. Use of hidden Markov models for QTL mapping. Johns Hopkins University, Department of Biostatistics Working Papers. 2006;125. Available: http://biostats.bepress.com/jhubiostat/paper125.

[34] Sen S, Churchill GA: A statistical framework for quantitative trait mapping. Genetics. 2001;159(1):371–387. 11560912 10.1093/genetics/159.1.371 PMC1461799

[35] Broman KW, Sen S. A Guide to QTL Mapping with R/qtl, New York: Springer; 2009.

[36] Churchill GA, Doerge RW. Empirical threshold values for quantitative trait mapping. Genetics 1994,138(3):963–971. 7851788 10.1093/genetics/138.3.963 PMC1206241

[37] Li H, Huang Z, Gai J, Wu S, Zeng Y, Li Q, et al A conceptual framework for mapping quantitative trait Loci regulating ontogenetic allometry. PLoS One. 2007;2(11):e1245 18043752 10.1371/journal.pone.0001245 PMC2080758

[38] Huang Z, Tong C, Bo W, Pang X, Wang Z, Xu J, et al An allometric model for mapping seed development in plants. Brief Bioinform. 2014;15(4):562–570. 23543351 10.1093/bib/bbt019

[39] Zhang WK, Wang YJ, Luo GZ, Zhang JS, He CY, Wu XL, et al QTL mapping of ten agronomic traits on the soybean (*Glycine max* L. Merr.) genetic map and their association with EST markers. Theor Appl Genet. 2004;108(6):1131–1139. 15067400 10.1007/s00122-003-1527-2

[40] SAS Institute Inc. SAS/STAT 9.3 User's Guide. Cary, NC: SAS Institute Inc 2011.

[41] Hanson C, Robinson H, Comstock R. Biometrical studies of yield in segregating populations of Korean Lespedeza. Agronomy J. 1956;48(6):268–272

[42] Hinkelmann K, Kempthorne O. Design and analysis of experiments, advanced experimental design, vol. 2 New Jersey: John Wiley & Sons; 2005.

