# Supplementary figures and images for: Composite Interval Mapping Based on Lattice Design for Error Control May Increase Power of Quantitative Trait Locus Detection
Source: PLoS One. 2015 Jun 15;10(6):e0130125. doi: 10.1371/journal.pone.0130125 (PMC4468128; doi:10.1371/journal.pone.0130125)

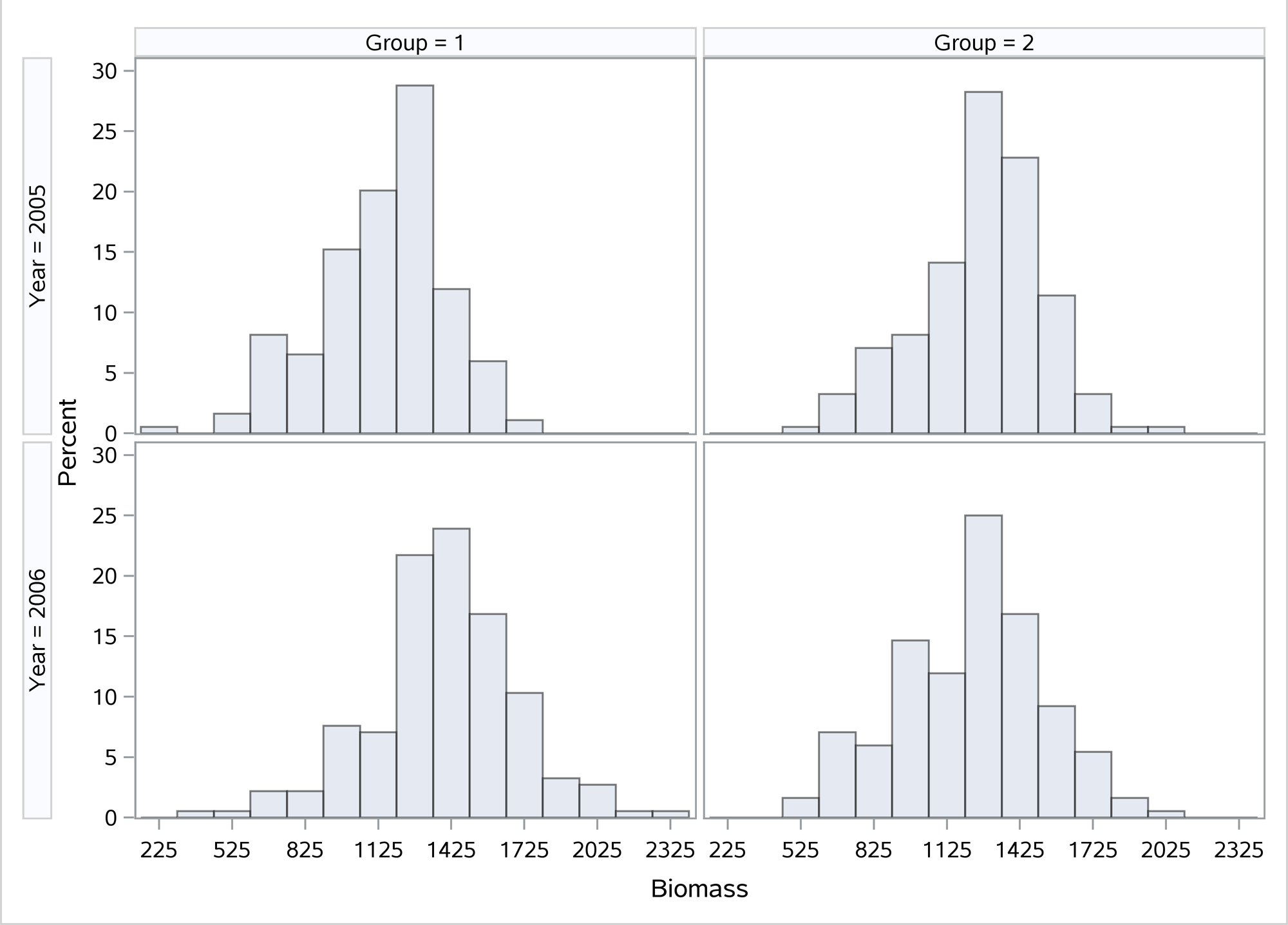

Supplement: S1 Fig — Biomass was measured in kg hm-2. (TIFF) [file pone.0130125.s001.tiff]
