# Supplementary material for: Composite Interval Mapping Based on Lattice Design for Error Control May Increase Power of Quantitative Trait Locus Detection
Source: PLoS One. 2015 Jun 15;10(6):e0130125. doi: 10.1371/journal.pone.0130125 (PMC4468128; doi:10.1371/journal.pone.0130125)
Supplement: S1 Table — (DOCX) [file pone.0130125.s002.docx]

**S1 Table. Analysis of variance for the biomass trait in a population of 184 soybean RILs**

| **Source of variation** | **DF** | **SS** | **MS** | ***F* value** |
| --- | --- | --- | --- | --- |
| Year | 1 | 1878707.79 | 1878707.79 | 13.75** |
| Replication (Year) | 2 | 4190372.98 | 2095186.49 | 15.34** |
| Block (Year, Replication) | 52 | 3951711.85 | 75994.46 | 5.15** |
| Line | 183 | 37210258.09 | 203334.74 | 13.78** |
| Line × Year | 183 | 6649377.18 | 36335.39 | 2.46** |
| Error | 314 | 4633936.19 | 14757.76 |  |

DF, degree of freedom; SS, type III sum of squares; MS, mean squares. ** Significant at a significance level of 0.01. The block-within-replication effect was considered to be random effect, all other effects were considered to be fixed effects.
